# Review on Generation and Characterization of Copper Particles and Copper Composites Prepared by Mechanical Milling on a Lab-Scale

**DOI:** 10.3390/ijms24097933

**Published:** 2023-04-27

**Authors:** Sebastián Salazar Sandoval, Nataly Silva

**Affiliations:** Facultad de Diseño, Universidad del Desarrollo, Avenida Plaza 680, Las Condes, Santiago 7610658, Chile

**Keywords:** copper particles, nanoparticles, microparticles, copper composites, ball milling, mechanical milling, mechanochemistry, high-energy ball milling, top-down, bottom-up

## Abstract

This review aims to expose mechanical milling as an alternative method for generating copper-based particles (copper particles (CuP) and copper composites (CuC)); more specifically, via a top-down or bottom-up approach, on a lab-scale. This work will also highlight the different parameters that can affect the size distribution, the type, and the morphology of the obtained CuP or CuC, such as the type of mechanical mill, ball-to-powder ratios (BPR), the milling speed, milling time, and the milling environment, among others. This review analyzes various papers based on the Cu-based particle generation route, which begins with a pretreatment step, then mechanical milling, its approach (top-down or bottom-up), and the post-treatment. Finally, the characterization methods of the resulting CuP and CuC through mechanical milling are also discussed.

## 1. Introduction

There is a growing interest in Cu-based materials due to the metal’s intrinsic properties, such as high electrical and thermal conductivity, resistance to corrosion, malleability, and anti-microbial activity. Cu-based materials present promising prospects in optics, conductive films, medicine, and manufacturing of anti-microbial materials, among others [1,2,3]. Furthermore, Cu is abundant and much less expensive than other noble metals in research, namely gold or silver, making its studies more affordable. Under that premise, Cu plays a fundamental and universal role on a daily basis. For example, Cu has been used in piping for water distribution due to its durability and anti-microbial activity. Additionally, it is the main conductive component in electric wiring. Notably, Cu-based materials became more prominent with the emergence of SARS-CoV-2, acting as coating agents and assessing anti-microbial activity on surfaces, preventing fomite transmission [4,5,6]. Although the anti-microbial efficacy of Cu surfaces has been extensively studied, Cu-based materials usually cannot be used in the as-received state. Thus, their further processing is necessary [7]. This downside can be overcome by obtaining such materials in a different format, such as copper particles (CuP), in micro- and nano-metric sizes, and copper composites (CuC), thus allowing its incorporation in diverse applications while also being cost-effective.

## 2. Generation of Copper Particles and Composites

The generation of CuP and CuC with different sizes and morphologies has been extensively reported by different methods, displaying their respective advantages and disadvantages [8,9,10,11]. The difficulty generating CuP can be attributed to their tendency to oxidize when exposed to air, thus making it imperative to use an inert gas atmosphere and surfactants to prevent oxidation [2,12]. While CuP can be generated using physical and chemical methods, eco-friendly and greener approaches have gained much interest in recent years [13,14,15].

**Figure 1:**
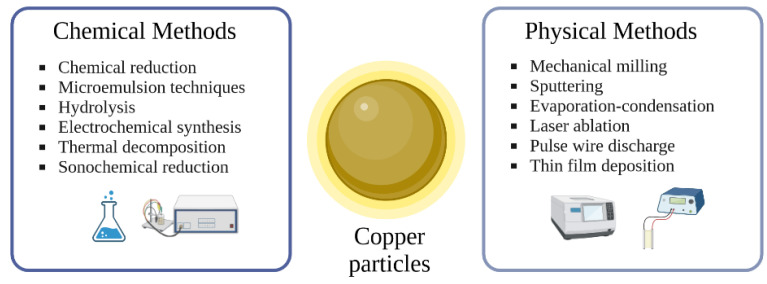
Generation routes of copper particle preparation [15]. Created with Biorender.com, accesed on 18 March 2023.

Some of the generation routes of CuP are summarized in Figure 1. The generation route will be a determinant in the physicochemical properties of the obtained CuP, such as size distribution, diameter, toxicity, stability, anti-microbial properties, and biological activity [15,16].

Among the physical methods for generating CuP or CuC, mechanical milling has been used to produce these materials as powders or grains [17,18,19]. The advantages of using mechanical milling as a generation route include forming finer particles without using hazardous solvents or high temperatures, controlling the degradation process, and the ability to homogenize otherwise immiscible materials. Furthermore, this method can also be used to treat waste and recycle materials to develop new composites or particles. Mechanical milling consists of reducing the metal precursor to a micro- or nano-metric size (top-down approach) or assembling larger structures due to the blending of the precursor salts (bottom-up approach) [20,21]. The mechanical mills can be grouped by their energy output, and the process can be carried out using conventional ball milling (CBM) or high-energy ball milling (HEBM). CBM is a powder-processing technique for reducing particle sizes and mixing different materials. This technique is widely used in mineral, pharmaceutical, and ceramic industries, as well as lab-scale synthesis. The HEBM technique is very different from CBM and was initially developed for producing new metastable materials, which cannot be made using thermal equilibrium processes [22]. In mechanical milling, kinetic energy is transferred to the precursor powder due to the collision between the former and the milling components (which can be a milling ball, for example), with the pressure and the impact generated by the milling media also playing a pivotal role in reducing or blending the starting material [21,23].

**Figure 2:**
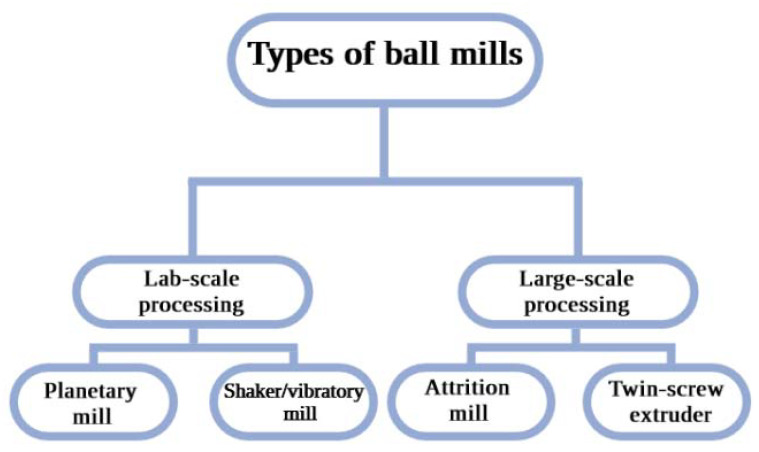
Classification of the most common types of ball mills according to their processing scales [26]. Created with Biorender.com.

Mills used for CuP or CuC generation are diverse, ranging from ball mills to drum ball mills, planetary mills, vibratory mills, and attrition mills [23,24,25]. Furthermore, types of mills can be classified according to their scale of processing. Figure 2 illustrates the classification of the most common types of ball mills. For large-scale processing, the continuous operation of attrition or vibratory mills and twin-screw extrusion are the most suitable. In turn, for lab-scale processes, planetary ball mills, shaker/vibratory mills, and CBM are the most used [26]. The scope of this review will solely focus on the generation of CuP and CuC on a lab-scale.

Mechanical milling can be categorized according to the changes that the precursor powders undergo [27,28,29]. The first category is mechanical activation, which occurs when the precursor remains chemically unchanged [24,26,30]. The second corresponds to mechanochemical reactions, where chemical changes are observed due to the influence of mechanical energy. Mechanical alloying can be classified into this group of processes [26,31]. Although their milling mechanisms are different, the parameters that can be altered to determine the particle size distribution of the obtained powder are usually the same. Such parameters include the ball-to-powder ratio (BPR), the diameter of the milling balls, the materials, and the shape of the milling media, as well as milling speeds, milling environments, the size of the milling media, the milling atmosphere, and the temperature [32,33,34]. The main components of the mill are the milling chamber and the grinding media (which can be milling balls or a vibratory disc, for example). Those components can also be optimized to obtain the desired outcome. The parameters to be considered when selecting a milling chamber are its material, volume, and filling degree, whereas the milling media should be made of the same material as the chambers to prevent the more rigid material from milling the softer one [26].

**Figure 3:**
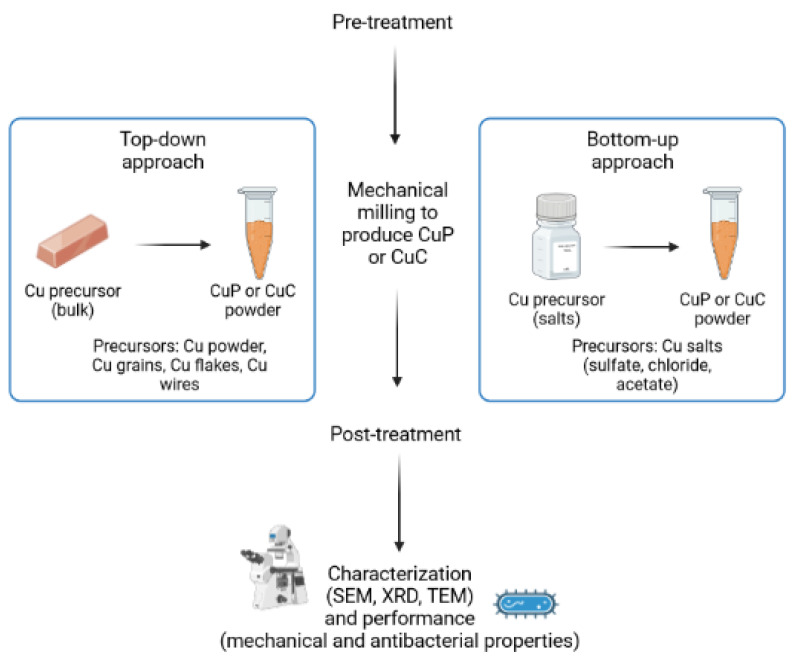
Generation route for the manufacturing of Cu-based particles through mechanical milling. Created with Biorender.com.

The milling conditions and the preparation method impact the properties of the obtained CuP, such as their average diameter or degree of crystallinity. Furthermore, treating the Cu materials before and after the milling also impacts the morphology and size of the resulting CuP or CuC. However, these steps are only sometimes discussed or performed by authors synthesizing Cu materials through mechanical milling. Figure 3 summarizes the CuP and CuC generation route, starting with pretreatment, then mechanical milling and its individual approach (top-down or bottom-up), post-treatment, and finally, characterization.

## 3. Pretreatment of the Starting Materials

The starting materials for the generation of CuP or CuC might require pretreatment before being subjected to the mechanical mill, according to the requirements of the equipment. Pretreatment methods of the starting materials can improve the efficiency of mechanical milling when synthesizing CuC or CuP [35,36,37].

Depending on the dimensions of the precursors and the requirements needed to conduct the milling conditions, the pretreatment can consist of polishing, grinding, sieving, or purification processes. Polishing and purification are usually performed by ultrasonication or an abrasive material (such as SiC) [38], and further immersing the Cu precursors into a solvent to remove impurities on their surface (NaOH, acetone, and toluene have been used for that purpose) [39,40,41]. Grinding is attained to improve the adhesion between Cu and other precursors to form an alloy, followed by treatment with an acid. The Cu precursors can also be pretreated by sieving, in case some mechanical milling equipment works better with specific sizes of starting materials.

Mechanical milling has also been reported as a pretreatment method to further develop a composite with a different technique or to subject the obtained Cu powders to a second milling with different conditions than the former [42,43].

Process control agents (PCA) are added to the Cu starting powder. The role of PCA is to prevent the adhesion of the precursors or the resulting powders to the milling chamber and media, the agglomeration or coarsening of the particles during the milling process, and excessive cold welding [22,40,44,45].

While authors have mostly reported using organic PCA, namely stearic acid, ethanol, toluene, methanol, and dichlorobenzene [40,41,46], inorganic PCA such as NaCl, NaOH, or NaSO_4_ can also effectively act as agglomeration inhibitors [12,47]. Note that the potentially corrosive effects on the milling media must be considered when choosing the PCA for the synthesis [44].

## 4. Synthesis of CuP and CuC Using Mechanical Milling

The following sections will discuss the top-down and bottom-up approaches to fabricating CuP and CuC.

### 4.1. Top-Down Approach Using Mechanical Milling

The top-down approach produces CuP and CuC by progressively breaking down and diminishing the Cu precursor dimensions to a micro- or nano-metric scale. The Cu- based particles synthesized through top-down approaches using mechanical milling are usually produced from Cu powders of high purity as the starting material [31,48,49,50]. However, other precursors, such as Cu flakes, grains, sheets, or wires, have also been reported [51,52,53].

Yadav et al. reported the synthesis and characterization of copper nanoparticles (CuNPs) from Cu powder (initial size of 200 nm, 99% purity) by wet milling [34]. The system consisted of a planetary mill, toluene as the medium, and combining two balls of different sizes (3 and 5 mm). The effects of the milling time on the CuNPs’ resulting size distribution were also tested. The particle size decreased as the milling time increased, obtaining a final crystallite size of 21 nm upon 40 h of milling.

Reduction of Cu powder to CuNPs was performed at cryogenic temperature by ball milling. A cryomill operated at 150 K under an Ar atmosphere was used for the developed system. Upon 3 h of milling, the as-synthesized CuNPs were dispersed in methanol and sonicated [33].

Amal et al. synthesized CuO nanoparticles from Cu powder (2–5 µm) using dry high-energy ball milling [54]. Different milling times, ranging from 4 to 16 h, were used to determine their effects on the final size of the nanoparticles.

The effects of HEBM on the particle size, morphology, and hardness of spherical CuP were evaluated by Ansell et al., in terms of milling time and BPR [55]. The processing of Cu powder without significantly compromising its physicochemical integrity is essential in additive manufacturing applications. HEBM was confirmed to be an excellent tool for Cu powder processing, without affecting the spheroidicity. The optimal BPR and milling times were determined to prevent the formation of Cu flakes, which are undesirable for additive manufacturing applications.

Bor et al. studied the morphological evolution of Cu precursors under planetary ball milling, by varying parameters such as the rotation speeds, ball diameters, and BPR [35]. They concluded that the milling conditions allowed them to control the synthesized Cu powders’ final particle size and morphology. The conclusions were supported by a ball motion simulation, where the milling parameters, namely force, energy, ball sizes, and rotation speeds, were tested.

Most articles describe that generating CuNPs through mechanical milling requires a prolonged time to reduce the Cu starting powder to nanometric size. However, by adjusting the milling conditions and reducing the milling times, it is also possible to obtain copper microparticles (CuMPs). Although CuNPs are often obtained with spherical morphology and monodispersity, CuMPs might present diverse morphologies, including sheets, flakes, or agglomerates.

Cryomilling has also been employed by Han et al. to develop CuNPs reinforced with graphene (GNPs), in which the ball milling process promoted the formation of two sizes of graphene in the composite, thus enhancing the strength and fracture elongation of the Cu-graphene system [56].

Cu-based powders were developed as protective coating materials using HEBM [45]. The Cu powder precursor (99% of purity) ranged from 5 to 20 mm in diameter and was subsequently reduced to particles within a micrometric range (average size of 200 µm). The obtained CuMPs were also sieved to obtain even finer particles.

Finally, the CuMPs were alloyed with Zn and Ni after heat treatment to obtain a nanocomposite that could exhibit synergic effects, inhibiting biofilms formed by pathogens. Cu-Zn composites and their stability were also studied in the work of Gamboa et al., which was processed using low-energy ball milling. Low-energy conditions were chosen in this study to promote the formation of a metastable phase. Pure Cu and Zn were used as precursors (99%), without previous treatment. The obtained Cu-Zn composite was compared with a Cu-Zn beta-phase alloy milled afterward [57].

A Cu-Ni-Fe-based alloy was prepared as an alternative for carbon-consuming anodes. The composite was prepared by HEBM, starting from pure Cu, Ni, and Fe (each of 99% purity). The ball milling was performed under an Ar atmosphere and stearic acid was added to the mixture to prevent excessive cold welding and the precursors’ sticking on the milling tools [58].

The reduction of Cu powder (7 µm in size) for the development of a CuW composite was reported by Dong et al., which was prepared by ball milling [59]. A drum ball mill operating at 300 rpm for 5 h was used, promoting the reduction of the Cu powder and the subsequent adhesion of W onto the surface of the CuNPs. Lu et al. also proposed a Cu-W alloy to enhance the mechanical and electrical properties compared to native Cu particles [60]. The Cu-W system was prepared using high-energy ball milling, obtaining a composite with high stability and increased conductivity, elongation, tensile strength, and stability.

A spinel phase of the copper-ferrite composite was developed by Marinca et al. by combining heat treatment and ball milling. Tenorite (CuO) and hematite (α-Fe_2_O_3_) were used as precursors [52]. Further, a single Cu spinel phase was obtained using heat treatment in ranges from 600 to 1000 °C. The synthesized CuFe_2_O_4_ was milled a second time to refine the composite’s crystallite size and to induce superparamagnetic behavior. A similar composite consisting of a Cu-Ni alloy was synthesized by Bettge et al., by ball milling, where the Cu-Ni particles exhibited a superparamagnetic nature, for a potential application in magnetic hyperthermia [61].

Salvo et al. reported a reinforcement of Cu with graphene. However, mechanical milling, hot pressing, and sintering were used instead of low-temperature milling and extrusion to disperse the graphene nanosheets on the Cu matrix. The matrix’s electrical conductivity, mechanical, and physical properties were reported and improved in comparison to pristine GNPs or Cu [46]. It was also reported that, as the content of graphene increased, the mechanical properties of the composite were reduced due to agglomerations of the former in the Cu matrix.

The incorporation of Al in a Cu matrix was also described by Saberi et al. using ball milling and PEG as a space holder. Notably, the Cu-Al matrix exhibited a porous nature, for its potential application as a filter, heat exchanger, or as a fuel cell. The Cu-Al composite presented a porosity percentage of 88% and microporosity, with the pore sizes ranging from 50 to 200 µm [62]. Abu-Okail et al. also proposed a Cu-Al composite with apparent porosity; however, graphene nanoplatelets were added to the mixture to enhance the mechanical properties of the nanocomposite [63].

Zirconia powder was used as a precursor to develop a Cu-ZrO_2_ composite produced by high-energy ball milling. Upon deposition of ZrO_2_, the hardness increased up to three times compared to the Cu precursor. It was concluded that the refinement of the powders by ball milling also contributed to the strengthening of the nanocomposite [64].

On the other hand, yttrium nanoparticles (in the form of Y_2_O_3_) were incorporated into a Cu matrix using multi-step ball milling. The nanocomposite exhibited a microhardness three times higher than that of free Cu, while also increasing its compressive strength, ductility, and conductivity [65].

The reinforcement of Cu materials has also been reported using Ag and reduced graphene oxides to develop a Cu-Ag-GNPs composite through the combination of spark plasma sintering and ball milling. Adding such materials to the Cu matrix significantly increased the relevant parameters, such as hardness, bending strength, and fracture surface, of the samples [66].

Spark plasma sintering has also been employed in conjunction with mechanical milling to synthesize a Ti-C-Cu composite. The properties of the system in terms of compressive strength, hardness, deformation, and conductivity were improved compared to pure Cu powder [67]. The effect of the carbon source on the structure of the final composite was evaluated, with nanodiamonds and carbon black promoting the faster reduction of the precursors during ball milling, in comparison to graphite.

Güler et al. proposed Ag and Al_2_O_3_ to reinforce Cu powder. Electroless silver plating and hot pressing were used for the deposition of Ag in the Cu-Al_2_O_3_ matrix, whereas mechanical milling was performed to produce the powder consisting of the Cu-Al_2_O_3_ composite [38]. In another study, Güler et al. also investigated the effects of the milling time on the coating thickness and composite morphology of Ag-Cu. Optimal bonding between Ag and Cu was attained upon 120 min of milling, obtaining a silver coating of 2 µm [47].

Although Cu powders of high purity are the precursor of choice for the synthesis of CuP and CuC using mechanical milling, the use of Cu starting materials in the form of waste has also been reported. Waste Cu chips have been processed using high-energy ball milling to obtain CuNPs, as reported by Ramesh et al. [23]. A milling time of 75 h was carried out in a solid state and inert atmosphere to prevent the oxidation of the product. Aldham et al. developed a synthesis of CuP using waste Cu grains as the precursor, using a vibratory disc mill as the means of pretreatment of the Cu grains, and further milled with a planetary ball mill to obtain CuMPs [50].

Nasimul et al. evaluated the effects of milling time on the obtained CuMPs using a waste Cu pellet and a planetary ball mill. The Cu pellets were milled using toluene as the process control agent, where the particle size decreased as the milling time increased. However, after 20 h of milling, the obtained Cu powder started agglomerating to form larger Cu microparticles [67].

A summary of relevant data concerning the synthesis and characterization of Cu-based particles, namely CuP and CuC, using a top-down approach through mechanical milling is presented in detail in Table 1.

### 4.2. Bottom-Up Approach Using Mechanical Milling

The bottom-up approach employs mechanical milling to achieve an assembly or self-assembly starting from a molecular or atomic precursor. Due to its low cost, CuSO_4_ is a common precursor in the bottom-up approach to obtain CuP or CuC. However, using Cu (II) salts such as CuCl_2_, Cu(NO_3_)_2_, CuS, or Cu(COO)_2_ in mechanical milling has also been reported. The size distribution and anti-microbial activity of the obtained Cu-based particles after ball milling were found to depend on the salt precursor.

A catalyst consisting of a mesoporous polymer embedded with CuNPs was proposed as a novel system for solvent-free reactions by Wang et al. [13]. The polymer-CuNPs composite was prepared in the solid state by ball milling, using Cu(NO_3_)_2_ as the Cu precursor. After milling for 12 h at 298 K, and upon pyrolization in a nitrogen atmosphere at 600 K, the proposed system was obtained.

A method for developing disc-shaped Cu flakes was reported by Jo et al. The green synthesis involved synthesizing CuNPs through a solvothermal method [36]. The obtained CuNPs were subsequently transformed into Cu flakes using vibratory milling. The resulting Cu flakes were sieved, washed with toluene, and separated with centrifugation.

CuO powders synthesized from two different precursors, namely CuSO_4_ and CuCl_2_, were reported by Javadhesari et al. [12]. The CuO nanoparticles were obtained by HEBM using NaOH and NaCl as a medium. The antibacterial activity of the CuO nanoparticles was evaluated, where the CuO NPs obtained from CuCl_2_ exhibited more antibacterial activity than its CuSO_4_ counterpart [12].

Mechanical milling has also been applied to reduce Cu sulfides, namely CuS and Cu_2_S, using Fe and Mg as reducing agents.

Calka et al. obtained elemental CuMPs using electric discharge-assisted mechanical milling, which attained the reduction of the Cu_2_S precursor after only 5 min of processing [68]. Furthermore, the mechanochemical reduction of CuS and Cu_2_S was investigated by Balaz et al. [26], who demonstrated the complete reduction of Cu_2_S to elemental Cu using a planetary mill after 360 min.

However, under the same conditions, a large amount of unreacted Fe was identified in the case of the CuS precursor. Reducing CuS to elemental Cu required longer milling times, leading to sulfide byproducts.

A CuNPs-carbon nanotube (CNT) composite was synthesized and characterized by Singhal et al. The composite was fabricated to reinforce and improve the mechanical properties of the Cu matrix [43]. A CuSO_4_ solution was added to a CNT suspension and further mixed using HEBM.

TiO_2_ nano-powders were deposited on a Cu matrix using HEBM, while also studying the effects of milling time, BPR, and the amount of Cu on the structure morphology and its anti-microbial activity against *E. coli* and *S. aureus* [69].

The synthesis and characterization of Cu-based particles using bottom-up approaches through mechanical milling are summarized in Table 2.

## 5. Post-Treatment of the Obtained CuP and CuC

Following the mechanical milling, CuP and CuC materials can undergo further treatment to achieve a desired size distribution, remove impurities from unreacted precursors, or provide the obtained Cu powder with additional properties by incorporating another metal or material to generate an alloy or composite. Among the protocols described for treating synthesized CuP or CuC, washing with an organic solvent followed by centrifugation is extensively used, primarily to re-disperse the synthesized Cu materials or to remove the excess solvents and process control agents used in wet milling [34,37,42]. Sieving is performed when a determined size distribution or ranges are needed, as the resulting powder might be a mixture of larger and finer particles. When producing a CuP, the metal precursors are refined using mechanical milling and further consolidated and homogenized using hot extrusion methods [56].

Hot extrusion processes are conducted in a vacuum to prevent matrix oxidation while applying temperatures over 900 °C and pressures in the MPa range of 100 to 1500 MPa [56,71]. Hot extrusion can also improve the strength, density, and hardness of the Cu matrix when developing a composite, while also decreasing its coefficient of friction and wear rates [56,71,72,73].

The post-treatment of a CuC can also be performed through spark plasma sintering processes or electroless Ag plating [21,43,51,59]. The composition of the CuC, as well as the coating thickness and distribution of the deposited metal over the Cu matrix, can be controlled with these techniques. Further, the sintered parts of the alloy are evaluated in terms of their mechanical strength using compression machines in quasistatic conditions [60].

## 6. Characterization Techniques of the Synthesized CuP and CuC

Determining the oxidation state of the generated CuC or CuP, their morphology, size distribution, and their optical, mechanical, and biological properties are of utmost interest in most analyzed articles.

### 6.1. X-ray Powder Diffraction (XRPD)

XRPD is a non-destructive technique used to ascertain the crystal structure of the synthesized Cu powders. Furthermore, relevant information such as crystallite size, lattice parameters, oxidation states, and strains can be determined.

The diffraction peaks of the obtained Cu-based materials allow for the indexation and, therefore, the identification of metallic Cu (Cu^0^) [23] or common Cu oxides in the form of CuO and Cu_2_O, which correspond to the Cu^2+^ and Cu^+^ oxidation states of Cu, respectively [48,54], as well as the presence of peaks that can be attributed to impurities from the precursors or the reducing agents [12,13].

Metallic Cu exhibits characteristic diffraction peaks at (111), (200), and (220) planes, which can be indexed to the face-centered cubic structure of Cu [37,42,69,72].

The most prominent diffraction peaks of CuO composites were observed at (002), (−111), (111), and (200), which correspond to the characteristic crystal structure of CuO in the monoclinic space group [2,12,74].

Cu_2_O particles show pronounced diffraction peaks at (110), (111), (200), (220), and (311) spaces, thus confirming the formation of a single cubic phase [54]. CuSO_4_, a common precursor in synthesizing Cu-based particles, presents a triclinic structure in its hydrated state.

### 6.2. Scanning Electron Microscopy (SEM) and Energy-Dispersive Spectroscopy (EDS)

SEM is used to analyze the surface morphology of synthesized Cu materials. EDS is an analytical technique that provides the elemental composition and chemical characterization of the Cu-based composites. SEM analyses allowed researchers to demonstrate that the milling parameters, such as BPR, milling time, or rotation speed, had an impact on the morphology of the obtained CuP or CuC [37,46,69,75]. The most prominent structures were agglomerates or spheres. However, a second milling and further processing of the powders promoted the formation of flake-like particles [36,42,47]. EDS and elemental mapping analyses indicated the presence of Cu and O in the Cu-based particles. In the case of composites, EDS provided information about the elemental distribution and the dispersion of the added materials in the Cu matrix, with Al, C, GNPs, CNTs, Ti, Zr, and Y being among the most used [50,63,65,67,69].

### 6.3. Transmission Electron Microscopy (TEM) and Selected Area Electron Diffraction (SAED)

Characterization using TEM provides information on the size distribution, morphology, and average diameter of the synthesized Cu materials. It also allowed ascertaining the interface between Cu and the materials used to generate a Cu composite. The size distribution obtained from TEM can also be compared to those obtained from the crystallite sizes determined using XRPD [33,35,54]. As previously reported, the as-synthesized Cu-based particles are usually in the nanometric range. However, adjusting the milling parameters allows for obtaining micrometric materials.

If the analysis is carried out using an HR-TEM, the lattice fringes of the Cu material can be detected, thus obtaining information regarding its crystallographic structure [76].

Moreover, SAED is a technique used alongside TEM or HR-TEM to evaluate the crystallinity of the CuP or CuC, the phase composition, and regions of differing phases, by analyzing the diffraction pattern generated by the electron beam interacting with the material, as reported in previous studies [73,77]. This technique is complementary to the XRPD characterization, which allowed, for example, to confirm the presence of Cu-Ti or Cu-Al phases in a composite [62,76].

### 6.4. UV-Visible Spectrophotometry (UV-Vis)

UV-visible spectra can be used to study the presence of Cu’s characteristic surface plasmon resonance in its nanometric state. Metallic CuNPs usually display an absorption peak at 575 nm [53]. CuO and Cu_2_O nanoparticles, however, display a solid blue shift in their absorption peak compared to that of CuNPs, ranging between 290 and 360 nm [15]. UV-visible spectra also provide information regarding the deposition of NPs on a Cu matrix. For example, the association of Ag nanoparticles onto the surface of a CuP can be confirmed by analyzing the presence of their characteristic plasmon band, usually found at 420–450 nm [42,47].

## 7. Evaluation of the Properties and Performance of the Obtained CuP and CuC

### 7.1. Mechanical Properties

Cu-based particles can be reinforced with a volume fraction of another metal using mechanical milling to modify or improve the mechanical properties, such as tensile strength, hardness, dislocation density, toughness, and fractography. CuC generated through mechanical milling displayed significant enhancements compared to the Cu matrix.

Cu powder reinforced with TiO_2_, GNPs, Al_2_O_3_, or graphene presented changes in its mechanical properties, with microhardness, elongation, toughness, tensile strength, and ductile fracture presenting the most noticeable improvements, up to 10 times in some cases [65,66,67,75]. These properties were measured using Brinell hardness testing machines, pliers, tensile and torsion testing machines, micro-indentation testing, and Oliver–Pharr methods [28,46,55,62,67,78].

### 7.2. Anti-Microbial Activity Assays

Due to increasing microbial resistance against a broad spectrum of anti-microbial agents, the study of CuP and CuC and their anti-microbial properties has become of great interest as an alternative to combat these resistant microorganisms. The anti-microbial activity of metallic Cu, Cu oxides, and CuC generated through mechanical milling has been investigated against Gram-positive and Gram-negative bacteria.

CuP and CuC generated through mechanical milling approaches exhibited high antibacterial activities. Smaller particles presented higher anti-microbial activities, depending on their morphology and size distribution. Even the Cu precursor had an impact on the anti-microbial effects. As a starting material, the CuO particles obtained from CuCl_2_ displayed higher anti-microbial efficacy than its CuSO_4_ counterpart [12]. Furthermore, metallic Cu particles generated using mechanical milling have exhibited microbial reduction up to 99% against both *E. coli* and *C. albicans* [48,69]. Notably, CuS microparticles generated through mechanochemical approaches were effective in both Gram-positive and Gram-negative bacteria [79,80]. CuC also showed anti-microbial properties, where Cu-GNPs and Cu-Ti composites were shown to be biologically active against *E. coli* and *S. aureus* [46,67].

## 8. Conclusions and Final Remarks

Mechanical milling allows for versatility and novelty in developing Cu-based particles. The obtained materials’ average size, type, and morphology can be controlled by adjusting the milling conditions, such as BPR, rotation speeds, and ball diameter. While Cu has interesting properties on its own, it is worth noting that incorporating elements such as graphene, carbon-based materials, Ti, Ag, or Al enhances the physicochemical properties of the Cu powder, where a synergic effect might occur. Mechanical milling has its shortcomings. Contamination can be generated during the mechanochemical processing, often from the abrasion of the milling media as well as from the atmosphere during the milling. Additionally, specialized equipment is needed to perform the reduction of the Cu precursors.

However, as the amount of energy is usually lower in comparison to related techniques to reach nanocrystalline materials, mechanical milling is undoubtedly a promising approach to develop a scalable synthesis of Cu-based particles, with potential applications in relevant fields, such as electronics, water remediation, sensors, pharmaceutical sciences, packaging, anti-microbial materials, and agriculture.

## Figures and Tables

**Table 1:** Summary of the top-down approach through mechanical milling methods for synthesizing Cu-based particles.

Pretreatment	Precursors	Type of Milling	Milling Conditions	Post-Treatment of the Obtained CuP or CuC	Morphology, Average Size	References
Toluene used as PCA to prevent oxidation and agglomeration	Cu powder (99% purity), initial size of 200 nm	Wet ball mill	40 h, 250 rpm. Ball size of 5 and 3 mm. Ball weight of 540 g. BPR 8:1	-	Agglomerates, 21 nm	[34]
The milling media cooled with liquid nitrogen	Cu powder (99% purity)	Cryogenic ball mill	Ar atmosphere, 3 h, 150 K. BPR 100:1	Washing with methanol, ultra-sonication for 15 min	Spherical, 30 nm	[33]
The waste Cu chips cleaned through ultrasonication	Cu chips (30 g) obtained from machining areas	Planetary mill	Ar atmosphere, 75 h, 400 rpm	-	Agglomerates, 50 nm	[23]
-	Cu powder (99% purity, initial size of 2–5 µm)	HEBM	4 to 16 h. Al_2_O_3_ balls of 1 mm. BPR 1:1	-	Spherical with some agglomerates, 100 nm	[54]
The precursors polished with SiC abrasive and further washed with acetone	Elemental Cu spheres (99% purity), 25 mm in diameter	Vibratory disc mill and planetary mill	Disc mill: 30 s, 1500 rpm. Planetary mill: 50 h, 300 rpm	-	Spherical, 0.25–1 µm	[38]
The precursors mixed in sealed quartz tubes under Ar atmosphere in a resistance furnace	Cu powder (99% purity)	CBM	Ar atmosphere, 177 rpm, 0.5 MPa. Rotating cylindrical steel cell: 200 mm/diameter; 29 mm/height. Seven balls of 25 mm, each of 67 g. BPR of 47.	-	Spherical, crystallite size of 5 nm	[57]
Stearic acid (0.5 wt.%) added as PCA	Cu powder (99.5% purity). Ni and Fe powders of similar purity added to the mixture to prepare a Cu-Ni-Fe composite	HEBM	Ar atmosphere, 40 h. Hardened steel vial of 55 mL, two balls of 14 and 11 mm, BPR of 2:1.	-	Spherical, 10–30 nm	[58]
The precursor powders washed with NaOH and submerged in acetone. Ethanol added as PCA	Cu powder (99% purity, 50 nm). Graphene nanoplatelets and Al_2_O_3_ added as reinforcement.	Planetary mill	2 h, 100 rpm. BPR 8:1	The obtained composite consolidated at 850 °C, under pressure of 850 MPa	Agglomerates, over 600 nm	[63]
-	Cu powder, initial size ranging between 4 and 7 µm	Ball milling	Drum mill, 5 h, 300 rpm. BPR 3:1	The CuNPs densified and mixed with W particles to generate a CuW composite	Spherical, 100 nm	[59]
-	Cu powder and stainless-steel powder of high purity (99%)	HEBM	2–60 min. 40 g of starting powder. Steel balls of 1.0 g each., BPR: 1:10, 1:5, 2:1, 1:1.	The milled powders cold-mounted and further polished	Agglomerates or flakes, depending on the milling times and BPR	[55]
Stearic acid (0.5 wt.%) added as PCA	Cu powder (99% purity, <75 µm average diameter). Graphene was also added to reinforce the Cu matrix	Planetary mill	Ar atmosphere, 4 h, 150 rpm, ZrO_2_ as grinding media, BPR 10:1	The consolidation of the powders by hot-pressing techniques	Agglomerates, flattened particles or flakes, depending on the milling time	[46]
The precursors physically mixed in a desired composition (71% Ni, 29% Cu *w*/*w*)	Cu powder (99% purity). Ni powder of similar purity was added to obtain a Cu-Ni composite	HEBM	2 h	The mixture placed in an Al_2_O_3_ crucible and heated at 1465 °C for 3 h	Spherical, 200 nm	[61]
-	CuO and α-Fe_2_O_3_ of 99% purity to obtain a CuO-Fe_2_O_3_ composite	HEBM	20 h. Agate balls, BPR of 11:1. Two milling processes under the same conditions were performed	-	Irregular shapes with some agglomerates, 30–50 nm	[52]
Stearic acid (2 wt.%) added as PCA	CuMPs (99%, <45 µm) and Al microparticles (99% purity, <45 µm) to develop a Cu-Al composite	Planetary mill	10 h, 300 rpm. BPR 10:1	The milled Cu-Al composite was further mixed with PEG, compacted (749 MPa), and sintered at 950 °C	From spherical to flake upon addition of Al to the Cu powder, microporosity	[62]
Stearic acid (3 wt.%) added as PCA	Cu powder (99% purity, 40 µm) and ZrO_2_ (99% purity) were used as raw materials to develop a Cu-ZrO_2_ composite	HEBM	250 rpm. Vertical milling machine, BPR 10:1	The composites mixed with paraffin to reduce the friction during compaction. Cold compaction at 700 MPa and further sintered at 950 °C for 2 h	Spherical large composites and flake-shaped particles	[64]
Methanol added as PCA	Cu powder (99% purity, oxygen-free)	Planetary mill	Ar atmosphere. A chamber of 250 mL	The Cu powder reinforced with Al_2_O_3_ by electroless Ag plater	Flake-like Cu clusters or Cu grains	[38]
Ethanol used as PCA	CuO (99% purity, <20 µm) mixed with Y_2_O_3_ particles (50 nm, 99% purity) to obtain Cu-Y_2_O_3_ composites	Planetary mill	1. Ethanol medium, 2 h, 200 rpm, BPR 15:1. 2. Ar atmosphere, 8 h, 250 rpm, BPR 15:1	The first obtained powder was reduced at 120 °C, the second milled powder was compacted and annealed by spark plasma sintering	Agglomerates, spherical upon second milling under the same conditions as the first	[65]
Methanol (0.25 wt. %) used as PCA	Cu particles (99%, average size of 61 µm)	Planetary mill	Ar atmosphere, methanol medium, room temperature, 300 min, 300 rpm. BPR 5:1,	A plating process was applied to obtain a uniform Ag layer on the CuP	Spherical after 60 min of milling; flake-like after 300 min	[47]
Stearic acid (1 wt.%) added as PCA	Cu powder (D_50_ = 10 µm) and WO_3_ (D_50_ = 50 nm) to obtain a Cu-W composite	HEBM	Ar atmosphere, 16 h, 650 rpm. BPR 10:1	The CuC was submitted to hydrogen atmosphere at 800 °C, then consolidated by hot pressing and sintered at 1000 °C for 2 h under 50 MPa	Mostly spheres or small agglomerates, 50–100 nm	[60]
The powder precursors annealed at 850 °C for 30 min to remove impurities	Cu powder (99% purity, average size of 40 µm) and Ti powder (98.5%, 15 µm) were used as precursors to obtain a Cu-Ti composite	HEBM	Ar atmosphere, stainless-steel balls of 8 mm, acceleration of 400 m/s^2^. BPR 18:1.	The CuC were sintered using spark plasma to obtain a Cu-Ti-C alloy. Graphite, nanodiamonds, and carbon black tested as carbon sources	Agglomerates, with black dots in the structure that represent the C source	[67]
The ball milling process modeled through discrete element model (DEM) simulations	Cu powder (99% purity, average size of 45 µm)	Planetary mill	48 h. CEBM (10, 50, 100 rpm) HEBM (300, 500, 700 rpm). Balls of 1 and 10 mm in diameter. BPR 10:1	-	The morphology changed from spherical to flake-like CuP as the milling time increased. The average size decreased upon increasing the rotation speeds (27 to 9 nm).	[35]

**Table 2:** Summary of the bottom-up approach methods for the synthesis of Cu-based particles.

Pretreatment	Precursors	Type of Milling	Milling Conditions	Post-Treatment of CuP or CuC	Morphology, Average Size	References
-	CuSO_4_ NaBH_4_/NaOH/reducing medium, EDTA was added as PCA	Planetary mill	3 h, 250 rpm. BPR 10:1	The CuNPs were mixed with CNTs, then dried at 60 °C for 2 h under vacuum	Spherical, 20–50 nm as CuNPs. Agglomerated as Cu-CNTs composites	[43]
The precursor was decomposed under inert atmosphere	Cu(HCOO)_2_	Ultrafine wet mill	240 min	The CuNPs milled in dipropylene glycol monomethyl ether (DMP) and dispersed in oleic acid	Spherical, 200–500 nm. Flakes, 2–5 µm	[42]
Dichlorobenzene added as PCA	Cu(COO)_2_	Vibratory disc mill	60 min. 45 g of 0.5 mm and 1 mm Zr balls.	The Cu flakes were sieved and further separated with toluene and centrifugation	Flakes and agglomerates, 100 nm	[36]
Quantum dots (QDs) were added to the copper solution, sonicated, atomized, and sintered	Cu(COO)_2_	Planetary mill	First ball milling (low): 6 h, 150 rpm. Stainless-steel jar. Second ball milling (high): 1 h, 300 rpm. BPR 10:1	The obtained QDs-Cu composite reduced at 300 °C for 5 h under Ar atmosphere	Spherical, 4 nm	[70]
Stearic acid added as PCA	Cu(NO_3_)_2_, glucose/reducing agent. Graphene added to form a reinforced Cu-GNPs matrix	Cryogenic planetary mill	4 h, 500 rpm, 193 K Steel milling balls of 6 and 10 mm, BPR 10:1	The mixed powders were cold-compacted, sintered in a tubular furnace, and further heated and extruded at 823 K	Cu-GNPs bars, 5 mm diameter	[13]
-	CuSO_4_ Formaldehyde/reducing agent. Al_2_O_3_ and GNPs added to obtain a Cu-Al_2_O_3_-GNPs composite	HEBM	20 h, 300 rpm. Al_2_O_3_ balls of 10 mm, BPR 50:1	The composite was compacted at 1200 MPa and further sintered at 1000 °C for 2 h	Spherical CuNPs, 50 nm. CuC, 700 nm	[53]
The precursors CuS and Cu_2_S were co-milled in a stochiometric ratio	CuS and Cu_2_S, Fe added as a reducing agent	Planetary mill	15–480 min, powder charge of 5 g, 250 mL tungsten carbide grinding chamber, 50 balls of 10 mm diameter, 500 rpm. Ar atmosphere	-	Grains, below 5 µm	[25]
NaOH or NaCl used as diluent phases	CuSO_4_ and CuCl_2_	HEBM	Air atmosphere, 1 h, 300 rpm. Stainless-steel balls of 10 and 7 mm. BPR 10:1	The obtained Cu powders were dispersed in an ultrasonic bath, washed with distilled water, centrifuged, and dried at 60 °C	CuONP synthesized with CuSO_4_: 14 nm; with CuCl_2_: 7 nm.	[12]
The precursors were mechanically pre-mixed in a conventional ball mill	Cu_2_S. Fe and Mg as reducing agents	Electric discharge assisted ball milling	5 min, Ar atmosphere. During milling, pulsed discharges travelled through the milling atmosphere and the precursor powders	-	Agglomerates, 5–100 µm	[68]
The precursors were mixed, and ethanol was added as PCA	CuSO_4_ (99%) and TiO_2_ (98%) were used to obtain CuNPs doped with TiO_2_	HEBM	10 h, 450 rpm, Zr balls of 3, 5, and 10 mm, BPR 10:1	The CuC was cooled, then dried, ultrasonicated, and ground	Average size of 65 nm. The average size of the Cu-Ti composite was proportional to BPR.	[69]

## Data Availability

Not applicable.
